# Grape Pomace: A Review of Its Bioactive Phenolic Compounds, Health Benefits, and Applications

**DOI:** 10.3390/molecules30020362

**Published:** 2025-01-17

**Authors:** Janice da Conceição Lopes, Joana Madureira, Fernanda M. A. Margaça, Sandra Cabo Verde

**Affiliations:** 1 Centro de Ciências e Tecnologias Nucleares (C2TN), Instituto Superior Técnico, Universidade de Lisboa, E.N. 10 ao km 139.7, 2695-066 Bobadela LRS, Portugal; janice.lopes@tecnico.ulisboa.pt (J.d.C.L.); joanamadureira@ctn.tecnico.ulisboa.pt (J.M.); fmargaca@ctn.tecnico.ulisboa.pt (F.M.A.M.); 2 Departamento de Engenharia e Ciências Nucleares (DECN), Instituto Superior Técnico, Universidade de Lisboa, E.N. 10 ao km 139.7, 2695-066 Bobadela LRS, Portugal

**Keywords:** grape marc, bioactive compounds, extraction methods, health benefits, agro-wastes valorization, sustainability, circular economy

## Abstract

The wine industry generates high amounts of waste, posing current environmental and economic sustainability challenges. Grape pomace, mainly composed of seeds, skins, and stalks, contains significant amounts of bioactive compounds and constitutes the main solid residue of this industry. Various strategies are being explored for its valorization, from a circular economy perspective. This review provides an updated overview of the composition of grape pomace from winemaking, highlighting sustainable methodologies for extracting phenolic compounds and their potential health benefits, including antioxidant, antimicrobial, antidiabetic, cardioprotective, antiproliferative, anti-aging, and gut health properties. Furthermore, this review explores the potential applications of this agro-industrial waste and its extractable compounds across the food, cosmetic, and pharmaceutical sectors.

## 1. Introduction

Grapes are a leading agricultural commodity, primarily cultivated for wine production. In 2022, the global grape production was estimated at 77,272,391 tons. The top five producers were China (20.2%), Italy (8.3%), France (8.0%), USA (7.7%), and Spain (7.7%) [1]. In the same year, 47.8% of the world’s grape production was used in wine production, with Italy (19%), France (17.5%), Spain (13.7%), USA (8.5%), and Australia (5.0%) being the top five wine-producing countries, accounting for nearly 63.7% of the global wine production [1]. In Europe, Italy (8.3%), France (8.0%), Spain (7.7%), Germany (1.6%), and Portugal (1.2%) together produced 26.8% of the world’s grapes. Furthermore, Europe generated 56.2% of the world’s wine, with the leading producers being the previously referred countries. The wine agro-industrial sector is vital to the Portuguese economy, with Portugal ranking as the ninetieth largest wine producer globally and the fifth largest in Europe [1].

Wine is considered a complex matrix composed of diverse quantities of both inorganic and organic compounds [2]. Moreover, the wine industry generates significant waste, including grape pomace, wastewater sludge, and yeast lees [3]. Typically, 20–30% of the weight of processed grapes becomes grape pomace, which mainly consists of skins, seeds, stems, and remaining pulp [4]. This poses both ecological and economic waste management challenges. Nowadays, effective waste management is mandatory, in a perfective circular economy, which aims to close resource loops and retain valuable materials within the economic system [5]. Poor waste management can significantly impact the environment, contributing to greenhouse gas emissions (CO_2_ and CH_4_) and the pollution of ground and surface waters. This is due to high biological oxygen demand (BOD) and chemical oxygen demand (COD)—with grape pomace COD ranging from 268 to 591 g O_2_/kg [6]. Disposal of grape pomace in landfills during harvest can also hinder biodegradation due to low pH [7]. Traditionally, grape pomace has been used for distilling spirits or used as livestock feed [7,8]. Grape pomace is a rich source of dietary fiber, residual sugars, lipids, proteins, phenolic compounds, and other essential bioactive compounds [9]. However, it has been reported that most animals cannot digest it efficiently [10]. Additionally, as a composting material, it lacks some essential nutrients, making it economically unviable [7]. Nevertheless, these same characteristics make grape pomace suitable for multiple valorization options. These range from extracting high-value compounds to producing energy or organic building blocks through intensive decomposition of organic matter.

Currently, multiple approaches are being investigated to exploit grape pomace, primarily focusing on extracting and valorizing its high-value compounds. Phenolic compounds, which are secondary plant metabolites, are among the most abundant bioactive compounds in grape pomace. They offer potential health benefits and have significant applications in the food, nutraceutical, pharmaceutical, and cosmetic industries. These components can be used as colorants, antioxidants, antimicrobial agents, or in packaging formulations [11,12,13]. In recent years, there has been an increase in studies aimed at developing strategies and techniques for recovering, valorizing, and applying these bioactive compounds [14,15,16]. The main challenge in the recovery process is to efficiently isolate target compounds from the grape pomace. This can be achieved through various conventional or emerging technologies, offering the potential to simultaneously recover multiple valuable ingredients and improve overall efficiency. This is achieved through a stepwise separation strategy that gradually moves from macroscopic to macromolecular, and finally to micromolecular scales [17]. Conventional extraction systems for valuable compounds from agro-food residues and other bioresources often involve the use of organic solvents and prolonged heating. However, these procedures can degrade the compounds of interest, making the process expensive, highly polluting, and often unsuitable for the intended purpose. Therefore, it is crucial to explore and optimize sustainable and effective extraction methodologies to maximize compound recovery, save energy, reduce the use of organic solvents, decrease environmental impact, and lower overall costs.

Researchers argue that it is feasible to recover essential bioactive compounds from grape pomace, making it a valuable by-product with promising industrial applications. Utilizing grape pomace and its compounds can lead to the creation of high-value products, significantly reducing waste and benefiting industries, society, and sustainability. In this context, this review summarizes the composition of grape pomace from winemaking and the sustainable methodologies for recovering phenolic compounds from it, highlighting their biological activities, health benefits, and applications across various industrial sectors. Overall, this review presents a comprehensive collection of grape pomace functionalities and outcomes, providing a systematic overview of its applications beyond segmented studies. Although some recent reviews have addressed the biological functions of grape pomace and its possible applications, most have focused on its use in the food sector. The novelty of this review lies in the comprehensive exploration of the intellectual properties related to this valuable residue’s applications in sectors rather than the food industry [18]. Therefore, this review represents a significant contribution to advancing sustainability and promoting a circular economy.

## 2. Methodology

This study employs an innovative review strategy grounded in a comprehensive retrospective analysis of research publications. It encompasses studies published from 2006 to 2024 that explored general characterizations while focusing on cutting-edge advancements in potential applications and emerging extraction technologies. To ensure a thorough and reliable literature review, data were sourced from reputable bibliographic databases and repositories, including Scopus, PubMed Central (PMC), PubMed, and editorial platforms such as ScienceDirect and MDPI. These sources were chosen for their extensive coverage of high-quality, peer-reviewed journals. The review process prioritized studies based on titles, abstracts, keywords, and publication years, with an emphasis on recent developments in the industrial utilization of grape pomace. A targeted search strategy was employed, incorporating Boolean operators (OR, AND), wildcard symbols (*, $), and a predefined set of keywords such as “grape pomace”, “grape byproduct”, “added-value compounds”, “grape pomace biotechnological applications”, “circular economy in the wine industry”, and “emerging extraction techniques”. Following the initial search, articles underwent a systematic screening process to ensure relevance and quality. This included removing duplicates, assessing abstracts, and, where necessary, a full-text review. For the patent search, the keywords “grape pomace”, “practical application”, “food application”, “cosmetic application”, and “pharmaceutical application” were used when searching the World Intellectual Property Organization (WIPOPATENTSCOPE) database. This strategy underscores the study’s commitment to identifying efficient valorization methods for grape pomace, aligns with contemporary principles of sustainability and innovation, and provides insights into its potential applications across industries.

## 3. Structure and Composition of Grape Pomace

Grape pomace is the main solid by-product formed throughout the pressing and fermentation phases of wine production. It comprises skins, seeds, and stalks remaining after winemaking, constituting 20–30% (*w/w*) of the processed grape weight. This percentage depends on grape variety, viticulture practices, environmental factors, and winemaking techniques [4,19]. All these variations in grape pomace composition represent a significant challenge for grape pomace valorization and fortification processing [20].

**Figure 1:**
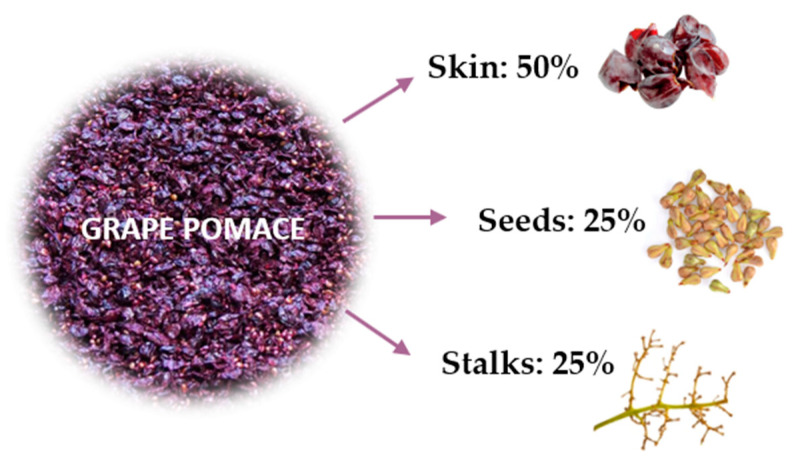
Solid compositions of grape pomace.

According to Jin et al. [9] grape pomace generated in wine production is composed of approximately 50% skin, 25% seed, and 25% stalks (Figure 1).

Grape skin exhibits a high concentration of fibers and sugars [9]. The fiber content varies from 51–56% by weight in red grapes to 17–28% in white grapes. Additionally, red grape skins are abundant in crude protein, fat, and ash (inorganic residue) [21]. Anthocyanins and tannins are also present in significant quantities, serving as valuable supplements for enhancing color development in wines [3]. According to Spinei and Oroian [22], grape skin comprises approximately 5% structural proteins, around 15% insoluble proanthocyanidins, 20% acidic pectic substances (of which, 63% are methyl-esterified), and 30% polysaccharides (cellulose, galactan, xylan, arabinan, xyloglucan, and mannan). In recent years, both red and white grape skins have garnered attention as potential sources of phytochemicals with numerous health-promoting effects.

Grape seeds primarily contain oil, comprising 8–20% by weight, consisting of a mixture of saturated and unsaturated fatty acids (such as oleic and linoleic acids), along with protein, non-digestible carbohydrates (cellulose and pectin), and significant amounts of phenolic compounds, rendering them highly nutritious and beneficial [3,9]. Despite the high market value, the oil is not usually recovered, likely due to challenges associated with separating the seeds from other pomace fractions [9]. According to Zanini et al. [23], the defatted seed residue comprises approximately 74.3% dietary fiber, 67.1% insoluble fiber, 31.5% lignin, 13.2% extractives in ethanol/benzene, 2.8% moisture, 8.8% protein, and 2.2% ash.

Grape stalks, the structural framework of the grape cluster, make up 25% of grape pomace by weight [9]. They are partially utilized as a source of astringent compounds, primarily represented by proanthocyanidins [24]. These stalks, mainly composed of lignified tissues, owe their structure to the abundant presence of fibers, like cellulose (12–36%), hemicellulose (14–26%), and lignin (23–34%) [25].

The physicochemical composition of grape pomace, as obtained from assorted studies, is outlined in Table 1. It should be noted that the variation in grape pomace components can be attributed to different factors such as grape variety, viticulture practices, environmental factors, winemaking techniques, and extraction techniques, among others. In general, the amount of phenolic compounds in red grape pomace is higher than that of white grape pomace, which is also affected by the different grape varieties [20]. Furthermore, Milella et al. [26] reported that grape pomace from cover crop soil presented higher concentrations of phenolic compounds and antioxidant activity than the other studied soil management techniques.

### 3.1. Extraction Methods to Recover Phenolic Compounds from Grape Pomace

**Figure 2:**
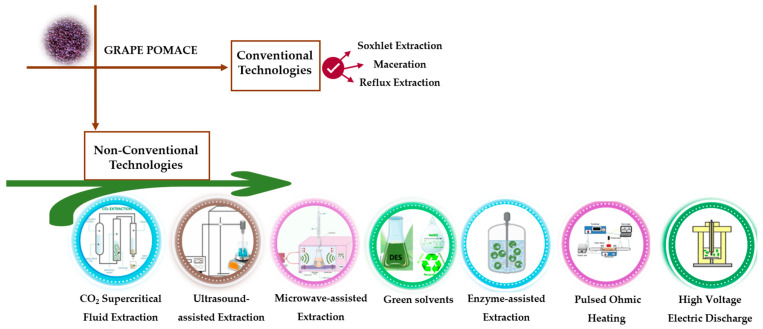
Extraction methodologies used to recover phenolic compounds from grape pomace.

The wine industry contributes significantly to environmental problems by disposing of enormous amounts of grape pomace directly into nature. Nevertheless, the valorization of this by-product can be dually beneficial by mitigating environmental harm and serving as a source of functional ingredients (particularly bioactive compounds). This approach aligns with circular economy principles, enabling the recovery of high-value compounds and their integration into economic systems [5]. Different extraction methodologies have been used to recover the bioactive compounds from grape pomace (Figure 2). From this perspective, it is conceivable to combine the extraction of valuable compounds with other processes, such as composting, which would further minimize the ecological footprint of winemaking. Despite the advancements, traditional extraction methods like maceration and Soxhlet extraction, face challenges, such as high energy demands, slow processing, and compromised sustainability, leading to continued disposal of large quantities of grape pomace [39]. To overcome these limitations, innovative and environmentally friendly extraction technologies are being explored to enhance the recovery of bioactive compounds from grape pomace, with improved efficiency, and using sustainable practices.

#### 3.1.1. Solid–Liquid Extraction

Solid-liquid extraction (SLE) relies on the effectiveness of various solvents or hot water extraction. Pintać et al. [40] investigated the efficacy of six solvents—acetone, ethyl acetate (EtOAc), 80% methanol (MeOH), 80% ethanol (EtOH), and 50% and 80% acidified MeOH—for extracting phenolic compounds and triterpenoid compounds from grape pomace. Among these, EtOAc exhibited the best results for extracting triterpenoid and phenolic compounds, such as flavanols, flavonones, stilbenes, and coumarins. Numerous factors contribute to the method’s efficiency, including the type of solvent, solvent-to-sample ratio, relative proportion of solvents, type of phenolic compounds being studied, particle size, temperature, and extraction time, among others [16,19,41]. Consequently, a comparison of the suitability of various tested methods thus far reveals that hydroalcoholic solutions yield a considerable amount of total extracts. Furthermore, they are considered more environmentally friendly and are recognized as safe by the European Food Safety Authority (EFSA) and the FAO/WHO Expert Committee on Food Additives [42,43]. The main challenges of SLE processes include low yields, lengthy durations, and volatile compound degradation. However, optimizing temperature and time can enhance solubility and diffusion while preserving phenolic stability for industrial scalability [16,40].

#### 3.1.2. Supercritical Fluid Extraction

Supercritical fluid extraction (SFE) is recognized for its environmental benefits, e.g., using supercritical carbon dioxide to achieve high selectivity and reduce solvent residues. This process operates under critical conditions (temperature and pressure) and exhibits solvency power and diffusivity similar to those of a liquid and a gas, respectively [19]. Hayrapetyan et al. [44] evaluated the potential of novel technologies such as supercritical carbon dioxide (SC-CO_2_) extraction processes for sustainable waste management with minimum environmental impact. The authors demonstrated that grape pomace could be valorized for the extraction of phenolic compounds and fibers, including pectic substances, by the SC-CO_2_ process, using water as a co-solvent [44].

#### 3.1.3. Ultrasound-Assisted Extraction

Ultrasound-assisted extraction (UAE) employs sound waves (20 kHz–10 MHz) to induce acoustic cavitation, enhancing cell wall disruption and solvent penetration [45]. According to Romanini et al. [46], ultrasound-assisted extraction showed improved results when compared to conventional extraction, extracting more than 11% of total phenolic compounds and 25% of total anthocyanins.

Panić et al. [47] evaluated the utilization of ultrasound-assisted extraction (UAE) combined with natural deep eutectic solvents (NADESs) to enhance polyphenol recovery from grape pomace. The produced grape pomace extract highlighted stability in NADESs as well as promising attributes such as enhanced bioavailability and eco-friendly characteristics. Also, these extracts are suitable for use in ready-to-use solutions in cosmetics, food supplements, and diverse food applications [47].

This method, recognized as an eco-friendly technology and a promising option for industrial scale-up, is effective for heat-sensitive compounds, providing high extraction efficiencies with minimal power usage and shorter processing times [45].

#### 3.1.4. Microwave-Assisted Extraction

Microwave-assisted extraction (MAE) is an eco-friendly technique that uses microwave energy (usually ranging from 300 to 550 W) to increase molecular movement, disrupt bonds, and improve the diffusion of bioactive compounds in the extraction solvent [48]. The type of solvent, extraction time temperature, microwave power, particle size, and solvent volume significantly impact the yield of anthocyanins from grape skin [45]. Álvarez et al. [14] demonstrated that the use of MAE significantly improves the recovery of polyphenols from grape pomace, increasing yields by 57%, anthocyanin content by 85%, cellular bioactivity by up to 133%, and dry product by 32%.

#### 3.1.5. High-Voltage Electric Discharge

High-voltage electric discharge (HVED) represents another slightly different, eco-friendly, effective, and innovative technique currently employed for the extraction of bioactive compounds from grape pomace. This method involves using high electric fields (40–60 kV/cm, 2–5 μs) to generate shock waves and cavitation, which fragment particles and disrupt cell walls for enhanced compound release [49]. The efficiency of this technique depends on the number of pulses, water-to-press-cake ratio, pH, and temperature [49]. According to El Kantar et al. [49], the energy of the HVED pre-treatment could be reduced by six times if the following solid–liquid extraction is performed in aqueous glycerol instead of water. This approach has shown promising results at the laboratory scale.

#### 3.1.6. Enzyme-Assisted Extraction

Enzyme-assisted extraction (EAE) is an eco-friendly alternative to traditional methods used for isolating bioactive compounds from grape pomace; this method involves replacing toxic and energy-intensive organic solvents with water as the primary solvent [50,51]. The enzyme-based extraction method uses specific enzymes to hydrolyze the polysaccharides in the plant cell wall, thereby decomposing the cell structure and releasing the bioactive compounds. Enzymes can selectively target and cleave specific bonds in plant materials and facilitate the efficient extraction of bioactive compounds; this results in higher yields and better purity of the extracted compounds [51]. Recent studies have documented the use of EAE for extracting phenolic compounds from grape pomace [50,51,52]. Stanek-Wandzel et al. [51] used advanced recovery techniques, such as enzyme immobilization, and integrated physical enhancement methods to significantly reduce production costs while maintaining or even improving the quality and bioactivity of phenolic extracts; this is considered a significant limitation that hinders the scalability of this EAE.

#### 3.1.7. Ohmic Heating

Ohmic heating (OH) is an effective method used to extract bioactive compounds from grape pomace; it involves combining electrical and thermal treatments using moderate electric fields and temperatures [41]. Studies have shown that the application of electric fields related to OH technology causes cell wall permeabilization and electroporation [41]. Moreover, OH-assisted extraction along with a hydroethanolic solvent could enhance the extraction of bioactive phenolic compounds from grape pomace residues [16]. Ferreira-Santos et al. [16] demonstrated that OH-assisted extraction provided a 40% increase in the total phenolic compound’s extraction when compared to a conventional heat treatment (without the application of electric fields) from grape pomace residues. This increment was accompanied by content upon the application of an electric field (37.98 ± 2.3 vs. 26.32 ± 1.4 mg CE/g dry GP, for OH and CH, respectively). Furthermore, OH extraction provided a 44% increase in flavonoid content and a 60% increase in the recovery of bioactive pigments from wine residues. Reference [16] also demonstrated the improved antioxidant capacity and selective action against cancer cells (Caco-2 and HeLa) of ethanolic extracts from grape pomace obtained using ohmic heating technology.

#### 3.1.8. High-Pressure Processing

High-pressure processing (HPP) is an emerging technology that applies high pressure (in order of MPa) to a matrix. This pressure causes air in the plant cell vacuoles to leak, damaging the cell membrane and allowing contact with the extraction solvent [50]. Teles et al. described how HPP in short durations (5 min; 200 MPa) seems to favor the release of proanthocyanidins. By altering cell structure through pressure-induced disruption of salt bridges and deprotonation of charged groups, HPP increases membrane permeability, enhancing the release of compounds [50].

#### 3.1.9. Deep Eutectic Solvents

The use of deep eutectic solvents (DESs) provides a sustainable, low-toxicity, and cost-effective alternative to extracting bioactive compounds from grape pomace, by utilizing heating (up to 80 °C) or lyophilization techniques. Panić et al. [47] used betaine/glucose as natural solvents to recover polyphenols from Graševina grape pomace. El Kantar et al. [49] used aqueous glycerol for the extraction of polyphenols from grapefruit peels, while Bubalo et al. [15] extracted phenolic compounds from the skin using DES with choline chloride and oxalic acid.

#### 3.1.10. Combination of Extraction Techniques

Some authors have explored the combinations of different technologies for extracting and isolating bioactives from grape pomace. Teles et al. [50] evaluated the effects of enzyme-assisted extraction and high hydrostatic pressure on the recovery of phenolic compounds from grape pomace. They highlighted that the combination of both technologies yielded the best extraction results, reinforcing their potential as sustainable and clean techniques used to recover bioactive compounds from food waste. Another study by Ferri et al. [17] aimed to optimize and validate the valorization of grape agro-waste for the production of bioactive molecules and new materials; the authors used solvent-based techniques and PLE, with the latter resulting in higher extraction yields. More recently, Panić et al. [47] reported on the use of ultrasound-assisted extraction combined with natural deep eutectic solvents to attain higher concentrations of polyphenols from Graševina grape pomace. The integration of both techniques demonstrated significant scalability and applicability for industrial use, as evidenced by the extracts’ collagenase-inhibitory effects.

Moreover, Caldas et al. [53] studied the effects of SLE (ethanol concentration) along with MAE and UAE; they observed that UAE was twice as efficient at extracting higher quantities of phenolic compounds. Some of the advantages and disadvantages of the extraction methods are listed in Table 2.

### 3.2. Effects of Extraction Conditions

As mentioned before, extraction stands out as an essential step for isolating and recovering bioactive compounds from grape pomace. Discrepancies in phenolic compound concentrations arise from factors such as soil and climate conditions, cultivation techniques, geography, harvesting dates, disease exposure, and extraction methods [38]. The selection of extraction techniques depends on the composition of the pomace, tissue type (leaves, stems, seeds, peels), and the properties of the target compounds. Typically, the goals of recovery strategies are to maximize the extraction yield, adapt to industrial demands, prevent degradation, and ensure sustainability and food-grade quality. Parameters such as solvent type, acidity, drying, and storage conditions play crucial roles in optimizing phenolic compound extraction [38]. A comparison using pressurized liquid extraction with pure ethanol, 50% ethanol, and acidified water as solvents indicated that 50% ethanol at 100 °C could extract a higher amount of total phenolic compounds, improving antioxidant activity [54]. As mentioned above, Pintać et al. [40] investigated the efficiency of six solvents (EtOAc, acetone, 80% MeOH, 80% EtOH, as well as 50% and 80% acidified MeOH) for extracting phenolic compounds and triterpenoid compounds from grape pomaces of different varieties. EtOAc yielded the richest polyphenol extract, 50% acidified MeOH was ideal for anthocyanin isolation, and acetone was optimal for extracting ursolic acid. For large-scale operations, 80% MeOH was preferred for high phenolic compound yields. Another investigation conducted by Moutinho et al. [55] studied the impact of solvent concentration (50%, 70%, 90% ethanol), temperature (20 °C, 40 °C, 60 °C), and pH (0.5%, 2.0%, 3.5% HCl) to extract bioactive compounds from grape pomace. The optimal conditions were identified as 70% ethanol at 60 °C and 3.5% HCl, which produced the highest phenolic compound concentration and antioxidant activity. Lower efficiencies were linked to 90% ethanol at 20 °C and 0.5% HCl. Pereira et al. [54] found that acidification enhances anthocyanin extraction but negatively affects other total phenolic content and antioxidant activity.

### 3.3. Bioactive Compounds from Grape Pomace

Phenolic compounds are the most abundant and widely distributed groups among the natural products present in plants; grape pomace is a rich source of these compounds [56]. These compounds are secondary plant metabolites, chemically characterized by an aromatic group containing one or more hydroxyl substituents [57]. In fact, grapes contain phenolic compounds in their seeds, skin, and short stems [58]. Phenolic compounds are categorized into different classes based on the similarity of their chemical structures and precursors. Ranging from simple to complex structures, phenolic compounds are divided into two main classes: flavonoids and non-flavonoids [59]. The predominant compounds include anthocyanins, along with hydroxybenzoic and hydroxycinnamic acids, flavan-3-ols (catechins and proanthocyanidins), flavonols, and stilbenes [59]. The total extractable phenolic content in grape pomace is approximately 60–70% in seeds, 30–35% in skin, around 5–8% by weight in seeds, and nearly 10% or less in pulp [60]. Furthermore, the composition of polyphenols varies across different parts of the grape. Grape skins primarily contain hydroxycinnamic acids, flavonols, flavonol glycosides, and anthocyanins, which are significantly influenced by the vinification method and contact time [57]. Grape seeds are rich in monomeric catechins and procyanidins (also known as condensed tannins) [61]. Other chemical classes such as phenolic acids, stilbenes, flavonols, hydrolyzable tannins, and organic acids have also been reported in grape seeds, but typically as minor components, differing according to variety or species [61]. Ferreira-Santos et al. [16] identified apigenin and naringenin as the major flavonoid compounds present in grape pomace derived from Italian wine production, with high levels of ellagic acid and *o*-coumaric acid. Moutinho et al. [55] identified 3-*o*-caffeoylquinic acid as the most abundant compound present in grape pomace from Portugal, followed by quercetin-3-*O*-rutinoside, quercetin-*O*-pentoside, and myricetin-*o*-rutinoside. Gallic acid, caffeic acid, syringic acid, vanillic acid, chlorogenic acid, and *p*-coumaric acid were also identified in ethanolic and acetone extracts from grape pomace from Portugal [62]. On the other hand, Peixoto et al. [63] reported petunidin-rutinoside, malvidin-rutinoside, β-Type (epi)catechin tetramer, quercetin-glucuronide, and *p*-coumaric acid hexoside as the main phenolic compounds present in extracts from Portuguese fermented grape pomace. In ethanolic extracts obtained from grape pomace of Argentina, the most abundant anthocyanins detected were malvidin-3-*O*-glucoside and malvidin-3-*O*-(*p*-coumaroyl)glucoside; the most abundant non-anthocyanins detected were (+)-catechin, (-)-epicatechin, syringic acid, and quercetin [35]. Furthermore, (-)-gallocatechin, (-)-epicatechin, and (+)-catechin were identified as the most abundant phenolic compounds in chardonnay pomace powder from the United States of America [36].

The composition of bioactive compounds of grape pomace, as gathered from diverse studies, is summarized in Table 1. It is important to note that the variability in bioactive components of grape pomace can be attributed to several factors. In recent decades, a significant research focus has been dedicated to studying the biological properties associated with polyphenolic compounds isolated from grape pomace.

## 4. Health-Promoting Effects of Phenolic Compounds from Grape Pomace

**Figure 3:**
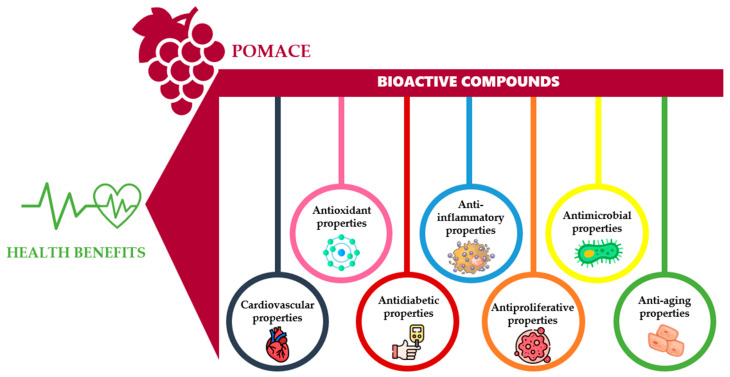
Effects of polyphenols isolated from grape pomace on human health.

The scientific interest in studying grape pomace phenolic compounds has grown in the last decades, given their potential beneficial effects on promoting human health. Phenolic compounds found in grape pomace induce actions that help boost health and reduce health risks, showing a wide range of biological properties such as antiallergenic, antimicrobial, anti-inflammatory, anti-aging, antitumor, antioxidant, anti-lipotropic, antithrombotic, cardioprotective, insulinotropic, and vasodilatory properties [27,64,65,66] (Figure 3).

### 4.1. Antioxidant Activity

The antioxidant activity stands out as one of the most noteworthy bioactivities of phenolic compounds extracted from grape pomace. Various methods have been employed to assess it. These methods include the 1-diphenyl-2-picrylhydrazyl (DPPH) radical scavenging activity assay, oxygen radical absorbance capacity (ORAC) assay, 1-crocin bleaching assay (CBA), ferric reducing antioxidant power (FRAP) assay, 2,2′-azinobis-(3-ethylbenzothiazoline-6-sulfonic acid) (ABTS) assay, thiobarbituric acid reactant substances (TBARS), and the Trolox equivalent antioxidant capacity (TEAC) assay [67].

The antioxidant effects of grape seed extracts were investigated in human keratinocytes exposed to UV radiation-induced oxidative damage. Pre-treatment with grape seed extract led to a notable reduction in reactive oxygen species (ROS) levels, particularly after infection [68]. Additionally, the antioxidant activity of white grape pomace was studied in human colonic epithelial cells exposed to H_2_O_2_-induced oxidative damage, revealing a significant reduction in ROS levels [69]. The antioxidant characteristics of extracts from grape pomace and grape pomace dietary fiber, both pre- and post-grinding, were evaluated based on their DPPH radical scavenging activity, ABTS diammonium salt radical scavenging activity, ferric reducing antioxidant power, and total phenolic content [70]. The findings suggested that micronized insoluble grape dietary fiber demonstrated increased ABTS radical scavenging activity, ferric-reducing antioxidant power, and total phenolic content compared to grape dietary fiber before and after grinding, although its DPPH radical scavenging activity was reduced. Furthermore, the grape dietary fiber components, along with the total soluble polyphenol content and antioxidant activity of a white grape (*V. vinifera*) variety were investigated [71]. The free radical scavenging activities of the former by-products were determined using the DPPH method, yielding EC_50_ values of 0.79 g (dry matter)/g DPPH (stem) and 1.32 g (dry matter)/g DPPH (pomace).

### 4.2. Antimicrobial Activity

Over the years, researchers have investigated the antimicrobial properties of grape pomace extracts and their ability to inhibit various pathogenic properties. Grape pomace phenolic compounds have shown the ability to inhibit the activities of enzymes and proteins found in bacteria. Although the reported findings are promising, the results vary regarding effectiveness and dosage. The antibacterial properties of phenolic compounds come from their ability to chelate iron or form hydrogen bonds with microbial enzymes and essential proteins [72]. For instance, grape tannins hinder microbial enzymes like peroxidase, pectinases, lactase, cellulases, and xylanases by altering their tertiary structures through non-covalent interactions with phenolic compounds. Similarly, grape flavonoids interact with proteins, potentially altering microbial interactions, and are considered the main phenolic compounds in grape pomace with antimicrobial potential [73]. They show synergistic effects with antibiotics and can inhibit virulence factors. Moreover, grape pomace polyphenols have been shown to affect the integrity of Gram-positive bacterial cell walls and disrupt the outer membranes of Gram-negative bacteria [38].

Karamati Jabehdar et al. [64] found that grape pomace extracts (1600 ppm) combined with 400 ppm of resistant starch effectively inhibited the growth of *Streptococcus* spp. Similarly, extracts from grape seeds demonstrated rapid inactivation of *Listeria monocytogenes*, where the absence of the environmental stress response regulon appeared to have played an important role in the resistance of *L. monocytogenes* to grape seed extract [74]. Ghendov-Mosanu et al. [13] discovered that grape pomace extract exhibited clear bactericidal activity against Gram-positive bacteria like *Bacillus subtilis* and *Staphylococcus aureus*, as well as some antibacterial activity against *Escherichia coli*. This antimicrobial effect was attributed to phenolic compounds, which can disrupt the cytoplasmic membrane’s integrity and inhibit nucleic acid synthesis in both Gram-negative and Gram-positive bacteria. Furthermore, synergistic interactions between grape pomace compounds (gallic acid, quercetin, luteolin, and protocatechuic acid) and several commonly used antibiotics (quinolones, β-lactams, tetracyclines, amphenicols, and fluoroquinolones) were observed to inhibit the growth of *S. aureus* and *E. coli* [72]. Sateriale et al. [75] assessed the antimicrobial and antibiofilm effects of grape pomace extracts on foodborne pathogens, including *E. coli*, *S. Typhimurium*, *L. monocytogenes*, *S. aureus*, and *Pseudomonas aeruginosa*. The extracts displayed significant antibacterial activity, with minimum inhibitory concentrations (MICs) ranging from 0.312 mg/mL to 5 mg/mL. The extracts were most effective against *E. coli* and *S. aureus*, with MICs as low as 0.312 mg/mL for *E. coli* and 0.625 mg/mL for *S. aureus*. Regarding antibiofilm activity, grape pomace extracts reduced biofilm formation by up to 90% in *E. coli* and *S. aureus* at a concentration of 0.5 mg/mL. The study also compared the antimicrobial potency of grape pomace extracts with standard antibiotics, including ampicillin. While the grape pomace extracts were less potent than antibiotics, they exhibited competitive antibacterial effects, particularly in disrupting biofilms, which are typically resistant to conventional treatments.

Grape stem extracts (195 mg/mL polyphenolic extracts in 10% dimethyl sulfoxide) were evaluated for their antimicrobial activity, inducing growth inhibition of Gram-positive (*S. aureus*, *L. monocytogenes*, and *Enterococcus faecalis*) and Gram-negative *(P. aeruginosa*) bacteria, which were preserved for 64 days [76]. Extracts from grape skins were tested against *L. monocytogenes*, *S. aureus*, *E. faecium*, and *E. faecalis*, as well as *Salmonella enterica* serovar Typhimurium and *E. coli*. Both organic and conventional extracts exhibited similar activity levels against all Gram-positive bacteria, but organic grape skin extracts were slightly more effective against *L. monocytogenes* due to their higher quercetin levels. However, neither extract inhibited the growth of the tested Gram-negative bacteria (*E. coli* and *S.* Typhimurium) [77]. Grape pomace extracts inhibited the growth of *E. coli*, *P. aeruginosa*, *Morganella morganii*, *E. faecalis*, methicillin-resistant *S. aureus*, *L. monocytogenes,* and methicillin-susceptible *S. aureus* at various concentrations (5−20 mg/ mL) [78].

Some studies have also explored the inhibitory potential of grape pomace extracts against fungi. Research on reducing fungal populations in wheat using grape pomace extracts highlighted their efficacy in inhibiting the growth of *Penicillium verrrucosum*, albeit showing less effectiveness against the *Aspergillus* genera [79]. Additionally, grape pomace extracts have demonstrated the ability to hinder the growth of *Zygosaccharomyces rouxii* and *Zygosaccharomyces bailii,* as well as *Candida krusei*, *Candida albicans*, and *Candida parapsilosis* [38,80,81]. Moreover, Corrales et al. [82] suggested the antifungal effects of grape pomace extracts on the growth of food-related molds, such as *Penicillium crysogenum*, *Penicillium expansum*, *Aspergillus niger,* and *Trichoderma viride*.

The antiviral properties of grape pomace bioactive compounds have been demonstrated, showing varying mechanisms and specificities. Early research on the antiviral properties of grape pomace bioactive activity by Konowalchuk and Speirs [83], showed that infusions and extracts could deactivate poliovirus particles. The active agents responsible for this deactivation were identified in the grape skin. Similarly, grape pomace extracts, at a concentration of 1 mg/mL, offered protection against influenza A viruses [84]. More recently, Zannella et al. [85] investigated the antiviral properties of Taurisolo^®^, a nutraceutical formulation derived from grape pomace polyphenols, against herpes simplex virus types 1 and 2 (HSV-1 and HSV-2). Taurisolo^®^ demonstrated significant in vitro antiviral activity, with low cytotoxicity confirmed by cell viability tests showing a safe concentration threshold below 800 µg/mL. Plaque assays revealed that Taurisolo^®^ effectively inhibited HSV-1 and HSV-2 at concentrations as low as 0.024 µg/mL and 0.097 µg/mL, respectively, in co-treatment and pretreatment experiments. The compound’s mechanism of action involved binding to viral particles, disrupting their envelope, and preventing entry into host cells. Real-time PCR confirmed the suppression of viral replication genes, and electron microscopy visualized the structural damage to the viral envelope, supporting its virucidal activity. Nevertheless, Taurisolo^®^ showed no activity against non-enveloped viruses like poliovirus, indicating specificity for enveloped viruses. These findings suggest that while certain grape pomace extracts may exhibit broad-spectrum antiviral effects, specific formulations like Taurisolo^®^ are targeted, offering promising therapeutic potential against enveloped viruses such as HSV [85].

### 4.3. Antidiabetic Activity

Diabetes affects a wide number of people around the world. Studying plant materials containing bioactive compounds and other phytochemicals capable of inhibiting the action of digestive enzymes (α-amylase and α-glucosidase) appears to be a viable approach to control this disease. High blood sugar levels indicate a medical condition called hyperglycemia, which is associated with type 2 diabetes [86,87]. At a concentration of 10 μg/mL, red and white grape pomace extracts could inhibit the activity of α-glucosidase enzyme by 63% and 43%, respectively, in yeast cells [37]. Comparable results were obtained for the alpha-glucosidase enzyme in the rat intestine, with inhibition activities of 47% for red grape pomace and 39% for white grape pomace. Additionally, post-prandial hyperglycemia was controlled in streptozotocin-induced mice after supplementation with 40 mg/100 g of red grape pomace extract [37]. Kong et al. [88] demonstrated that aqueous grape seed extract from Chardonnay grapes inhibited α-glucosidase more effectively than acarbose, a commonly used antidiabetic drug. The extract had an inhibitory concentration of 25.25 ± 0.53 μg/mL, compared to 1256.24 ± 1.3 μg/mL for acarbose.

It has been reported that certain phenolic compounds present in grape pomace, such as peonidin-3-*O*-acetylglucoside, quercetin-3-*O*-glucuronide, isorhamnetin-3-*O*-glucoside, and catechin, can block the active site and inhibit both salivary α-amylase and porcine pancreatic α-amylase enzymes through a competitive effect [87]. Increased expression of glucose transporter protein 4 (GLUT4) and peroxisome proliferator-activated receptor gamma (PPARγ) in adipose tissue, as well as the modulation of adipogenesis and an increase in adipose glucose uptake, were described by Costabile et al. [86]. Insulin sensitivity was enhanced in participants of this study after consuming a 250 mL beverage containing red grape pomace, in a randomized, controlled human clinical investigation. Similarly, gallic acid, another phenolic compound abundant in grape pomace, was highlighted by Variya et al. [89] for its antidiabetic effects. In vitro studies with 3T3-L1 adipocyte cultures revealed that gallic acid (2–20 μM) stimulated glucose transporter type 4 (Glut-4) translocation by activating protein kinase B (Akt) phosphorylation and the peroxisome proliferator-activated receptor gamma (PPAR-γ). These mechanisms enhanced glucose uptake, lowered blood glucose levels, and increased insulin sensitivity. Interestingly, in vivo experiments on a murine model showed that daily oral administration of gallic acid (100 mg/kg) over 42 days reduced body weight, contrary to the compound’s association with adipogenesis. In a clinical study, Taladrid et al. [90] explored the impact of grape pomace seasoning on 17 patients with high cardiovascular risk. Participants consuming 2 g/day of grape pomace seasoning exhibited significant reductions in blood pressure and fasting blood glucose levels (*p* < 0.05), demonstrating grape pomace’s hypotensive and hypoglycemic properties. These findings emphasize the therapeutic potential of grape pomace compounds in managing diabetes and improving metabolic health.

### 4.4. Cardioprotective Activity

Cardiovascular disease arises from alterations in fatty acid metabolism and excessive lipid peroxidation of low-density lipoprotein. Oxidative stress plays a crucial role in the pathophysiology of cardiovascular diseases and serves as a major marker for therapeutic interventions [91]. Tomé-Carneiro et al. [92] investigated the impact of a grape supplement on oxidized low-density lipoprotein, apolipoprotein-B, and serum lipids in statin-treated patients through a triple-blind, randomized, placebo-controlled trial for primary cardiovascular disease prevention. This grape extract reduced atherogenic markers and potentially provided additional cardioprotection beyond standard medications for primary cardiovascular disease prevention. In vivo assays on Wistar rats confirmed the potential of grape pomace extracts to reduce lipids (cholesterol, very-low-density lipoprotein, and triglycerides), suggesting grape pomace as an affordable alternative for treating coronary heart disease [65]. Fresh and fermented pomace from red grape varieties, rich in anthocyanins and condensed tannins, exhibited similar effects against isoprenaline-induced infarct-like lesions by suppressing cardiovascular enzyme markers. Furthermore, it demonstrated the downregulation of oxidative stress markers, particularly malondialdehyde, and the enhancement of serum antioxidant levels [93].

Similarly, Sanz-Buenhombre et al. [94] investigated the effects of Tempranillo grape pomace extract on low-density lipoprotein (LDL) regulation using intestinal (Caco-2) and liver (HepG2) cell models. Their findings suggested that the extract may contribute to lowering LDL levels by enhancing the expression of the LDL receptor (LDLr), which facilitates the removal of cholesterol from the bloodstream. Additionally, the study observed a decrease in the expression of the CYP7A1 gene, which encodes the enzyme involved in cholesterol catabolism. This effect was notably significant only after simulating gastrointestinal digestion, where gallic and vanillic acids appeared to play a role. These results suggest that metabolites generated during digestion or intact compounds from the grape pomace extract may help modulate cholesterol metabolism, offering potential cardioprotective benefits. Pomace from different grape varieties, containing significant phenolic compounds such as catechin, quercetin, kaempferol, trans-cinnamic acid, rutin, malvidin, and delphinidin, also showed a cardioprotective role at concentrations ranging between 0.25 and 2 mg/100 g of phenolic compounds [95].

Choleva et al. [96] evaluated the key factors involved in the platelet-aggregation process, including the inhibition of adenosine diphosphate and thrombin receptor-activating peptide. The authors reported that anti-platelet activity was not dependent on grape variety but rather strongly correlated with the extraction solvent utilized for extract recovery. Ethanolic extracts were more effective as anti-aggregation agents, with IC_50_ values of 162.1, 181.2, and 156.3 µg of the extract against the platelet-activating factor, adenosine diphosphate, and thrombin receptor-activating peptide, respectively. Additionally, the authors suggested that this functional property might be more related to the presence of specific phenolic compounds (e.g., catechin, malvidin-3-*O*-glucoside, quercetin, and epicatechin) and other micro-constituents than to the total phenolic content in the grape pomace extracts. Muñoz-Bernal et al. [97] indicated that monomeric and oligomeric flavan-3-ols compounds present in grape pomace could alter the platelet aggregation mechanism; they suggested that this functional attribute is more closely linked to the individual phenolic compounds than to the overall phenolic content of the grape pomace extracts.

### 4.5. Antiproliferative Activity

Despite considerable progress in basic research and clinical studies, cancer remains one of the most progressive and harmful diseases worldwide [66]. Recently, researchers have demonstrated the anticancer properties of grape pomace phenolic compounds, showing their effectiveness in vitro and in vivo, as well as their bioactivity in various cancer cells.

Phenolic compounds of grape pomace are known to inhibit proteases, drug-metabolizing enzymes in phases I and II; they can also interfere with metabolic pathways related to invasion, angiogenesis, and metastasis, alter cell-cycle checkpoints and apoptosis, and block receptor-mediated functions [98]. A study on cell lines suggested that grape seed proanthocyanidins could inhibit cancer cell invasion in a dose-dependent manner at concentrations of 10, 20, and 40 μg/mL [99]. Balea et al. [100] evaluated the antiproliferative effects of fresh and fermented grape pomace extracts from *Vitis vinifera* L. varieties. The authors observed that these extracts inhibited the proliferation of human breast adenocarcinoma (MDA-MB-231), human lung carcinoma (A549), and murine melanoma (B164A5) cell lines at 1 mg/L after 48 h of incubation, correlating this effect with the presence of phenolic compounds like isoquercitrin and quercetol. These findings were further supported by Spissu et al. [66], who found that phenolic-rich extracts of grape pomace reduced the viability of B16F10 metastatic melanoma cancer cells by 25 to 50% compared to the control, with effects depending on dose, treatment time, and extract origin. Pérez-Ortiz et al. [101] reported that grape pomace extract exhibited antiproliferative properties at concentrations ranging from 5 to 250 μg/mL in colon cancer cell lines (Caco-2, HT-29) and fibroblasts. This study demonstrated that grape seed extract has antitumor activity by downregulating Myc gene expression in HT-29 cells and upregulating Ptg2 in Caco-2 cells. The non-anthocyanin fraction of grape seed extracts also showed potential activity against colorectal cancer cells.

Under in vitro conditions, low doses of proanthocyanidins from grape seeds inhibit the growth of liver (HepG2) and cervical (HeLa) cancer cells [102]. By modulating the redox balance, grape seeds help inhibit cancer development through both pro-oxidant and antioxidant actions [102]. The authors demonstrated that grape stem extracts prevent ROS-induced DNA damage and suppress cervical and liver cancer cell growth. Their investigation into the antioxidant potential of these extracts showed that they could hamper the proliferation of these cancer cells at low concentrations, concluding that grape stem extracts have similar activity to grape seed extracts. Sanchez-Martin et al. [103] evaluated the cytotoxic effects of five phenolic compounds, including gallic acid, on colon cancer cell lines. Only gallic acid showed growth inhibition, inducing cell cycle arrest and nucleolar stress. In vivo, gallic acid inhibited tumor growth in a mouse xenograft model by stabilizing G-quadruplexes, leading to the downregulation of proto-oncogenes such as CMYC, commonly overexpressed in colorectal tumors.

The effects of grape pomace supplementation on colorectal cancer were explored in a study by Tian et al. [104], where mice with induced colorectal cancer were fed a grape pomace-rich diet. The results showed a reduction in disease progression, demonstrated by a significant decrease in tumor size and less colon epithelial damage, suggesting a protective role of grape pomace. The study also reported a decrease in cancer cell proliferation, marked by lower levels of β-catenin protein (a transcription factor), reduced cyclin D1 gene expression (a cell cycle protein), and increased p53 protein expression, which induces cell cycle arrest. Additionally, overexpression of anti-inflammatory proteins, such as TNF-β, and reduced expression of pro-inflammatory proteins like TNF-α, IL-1β, p65, and phosphorylated p65, contributed to the observed reduction in symptoms. The study also highlighted grape pomace’s protective effects on DNA hypermethylation. According to Hamza et al. [105], grape seed extract may exert antiproliferative effects by reducing inflammation, increasing apoptosis, and blocking cell growth in hepatocarcinoma. In addition, Luo et al. [106] reported that phenolic compounds from Cabernet Sauvignon and Muscadine grape pomace suppressed the growth of human metastatic breast cancer cell lines, with Muscadine extracts showing more significant effects, reducing cell viability by 95%.

### 4.6. Anti-Aging Activity

Polyphenols are substances often associated with cosmetics due to their high antioxidant power. This counteracts the formation of free radicals, combating external factors that undermine the health and beauty of the skin and thereby blocking premature skin aging [69,107]. Polyphenols play a critical role in maintaining skin integrity and resilience by protecting it from ultraviolet radiation, pollutants, and environmental stressors while preserving the lipid barrier and protein structures under oxidative and physical challenges [108,109]. Numerous studies have focused on grape pomace polyphenols to identify phytochemicals and potential applications of bioactive compounds for skincare products, resulting in high-value ingredients suitable for cosmetic formulations. Porto et al. [108] showed that linoleic acid, which makes up 72% of grape seed oil, exhibited anti-inflammatory and anti-aging effects when used in facial skincare products.

Draghici-Popa et al. [107] optimized a grape marc extract to enhance the photoprotective effect of sun protection creams, demonstrating the compatibility of the polyphenolic extract with the lotion base used, indicating an in vitro-estimated sun protection factor of about 10–15.

Cosmetic applications, beyond their antioxidant potential, have demonstrated that gallic acid in grape pomace effectively inactivates the proteolytic enzymes collagenase and elastase. These enzymes are responsible for the degradation of dermal protein structures, confirming the suitability of these polyphenols for anti-aging cosmetic preparations [110]. Additional studies on the anti-aging properties of grape pomace polyphenols have explored their potential benefits for the central nervous system. Grape seed extract has been shown to modulate age-related oxidative DNA damage and lipid peroxidation in the central nervous system of rats [111,112]. Aged rats exhibited improved memory performance, reduced production of reactive oxygen species, decreased carbonyl protein levels, increased thiol levels, and reduced hypoxic–ischemic brain injury. Resveratrol, a nonflavonoid polyphenol naturally found in red wine and grapes, has demonstrated neuroprotective effects against seizures, ischemia, and neurodegenerative diseases [113]. Zhang et al. [113] elucidated the molecular mechanisms underlying resveratrol-mediated neuroprotection. They showed that resveratrol protected dopamine neurons from lipopolysaccharide (LPS)-induced neurotoxicity in a concentration- and time-dependent manner by inhibiting microglial activation and reducing the release of proinflammatory factors. Mechanistically, this neuroprotection is attributed to the inhibition of NADPH oxidase.

### 4.7. Gut Health Activity

The gut microbiota continues to serve as an indicator of intestinal health. Research has delved into the effects of prolonged supplementation with grape pomace extracts rich in phenolic compounds on the intestinal microbiota of rats. According to Chacar et al. [114], feeding at concentrations of 2.5 and 5 mg/kg/d has specifically promoted the proliferation of gut bacteria. An in vitro simulation of gastric and intestinal digestion of grape pomace extracts indicated to inhibition of the growth of potential pathogenic bacteria and the promotion of the growth of probiotic bacteria [115]. Additionally, phenolic compounds, and more importantly, flavonoids, have been reported to function as prebiotic components. Furthermore, Casanova-Martí et al. [116] observed an upward trend in the growth of Bacteroidetes and a downward trend in Firmicutes after supplementing rats with grape seed proanthocyanidins. Rollová et al. [117] indicated that *V. vinifera* cane extract from plant waste induced metabolic activity as well as total biofilm biomass production in probiotic strain *L. acidophilus* LA-5, while inhibiting the growth of opportunistic microorganisms *C. freundii* DBM 3127 and *E. coli* DBM 3125.

Touriño et al. [118] identified metabolite compounds that interact with colonic epithelial tissue after the ingestion of grape dietary fiber. Their findings revealed that proanthocyanidin oligomers and polymers are depolymerized into (epi)catechin units during transit along the digestive tract. After ingesting grape dietary fiber, free (epi)catechin and its conjugates, along with free and conjugated microbially-derived phenolic metabolites, encounter the intestine epithelium for more than 24 h, potentially contributing to positive effects on gut health. Maurer et al. [119] highlighted that the polyphenols associated with the dietary fiber of grape peel powder seemed to reestablish the intestinal barrier function, whereas fiber-bound phenolics could restore cecal metabolism to produce beneficial metabolites.

## 5. Applications of Grape Pomace Bioactive Compounds

This review addresses emerging extraction processes and their effects on the recovery of bioactive compounds. It is noteworthy that the period between grape pomace production and its valorization, along with recovery procedures, directly impacts phytochemicals and, consequently, the potential of bioactive compounds. As grape pomace began to be a focus for modern industries, its valorization offered alternatives to minimize the environmental impact of winemaking activities promoting the circular economy. A suitable recovery strategy is crucial for the effective utilization of grape pomace, ensuring the safe and high-quality recovery of compounds. The applications of bioactive compounds from grape pomace have been reported in the literature for various food, cosmetic, and pharmaceutical industries as versatile ingredients or additives, making this a promising field.

To visualize the technological development and novel trends in the industrial application of grape pomace, an analysis was performed using the world intellectual property database “WIPO-PATENTSCOPE” (accessed on 23 October 2024). The patents shown in Table 3 involve the use of grape pomace either as a primary component or as an auxiliary in various development processes. The focus includes the production of new bioactive food products and beverages, the generation of protein–polyphenol complexes, the production of initial materials for other food products, and the enrichment of animal feed. Furthermore, patents are also related to innovative pharmaceutical formulations used to treat neurodegenerative, cardiovascular, or cerebrovascular diseases, and for skincare preparations.

### 5.1. Food Industry

The bioactive compounds from grape pomace, particularly polyphenols, have multifunctional characteristics that make them suitable for developing novel and functional foods. Numerous studies have explored the progress that has been made in incorporating grape pomace extracts directly into food products, leveraging grape pomace as a polyphenol carrier. This approach increases the concentration of phenolic compounds and enhances antioxidant potential, offering clear benefits for consumer health and food quality preservation during storage.

The use of grape pomace extracts and grape seed flour in various food products such as confections and bakery items has significantly enhanced the antioxidant potential of these products and opened up interesting prospects for the use of medical gastronomy or personalized gastronomy [120]. For instance, biscuits enriched with grape pomace extract showed higher total phenolic content compared to control wheat biscuits and demonstrated greater antioxidant activity against DPPH and ABTS radicals [121]. Similar results were observed when aqueous extracts of grape pomace replaced water in bread formulations [122]. Recent studies have shown that bread enriched with 5% and 10% grape pomace extracts displayed increased ferric-ion-reducing antioxidant power compared to traditional bread, even after undergoing in vitro digestion [123,124]. Additionally, phenolic compounds recovered from grape seed flour have been used to enrich noodles, cereal bars, and pancakes, offering high antioxidant activity and consumer acceptance [125].

Ratu et al. [126] examined the effects of incorporating grape skin powder into cheese formulations; they found that a minimum inclusion of 1.6% grape pomace is typically needed to boost the antioxidant activity in Caciotta cheese.

Deolindo et al. [127] investigated the incorporation of polyphenol-rich extracts from the skin and seeds of *Vitis labrusca* (Bordeaux grapes) into Petit Suisse cheese. Hydroalcoholic extraction yielded extracts with high antioxidant activity, with grape seed extract showing the highest level at 3637 ± 4 mg (ascorbic acid equivalent)/100 g, followed by grape skin extracts at 1033 ± 10 mg (ascorbic acid equivalent)/100 g. The fortified cheese exhibited increased phenolic content and antioxidant activity, largely retained during storage, with only a slight decline after 28 days. Sensory evaluation revealed a 73% acceptance rate, despite minor alterations to texture, flavor, and appearance. Functionally, cheese enriched with grape seed extract inhibited 77% of angiotensin-converting enzyme activity, suggesting antihypertensive potential, compared to 38% inhibition by cheese containing only grape skin flour. Gaglio et al. [128] incorporated 1% (*w/w*) of grape pomace powder into fresh ovine “Primosale” cheese and evaluated its effects on the cheese’s nutritional and functional properties. Using selected *Lactococcus lactis* strains, the enriched cheeses had increased protein content and antioxidant activity during simulated digestion, alongside reduced fat and slightly elevated lipid oxidation. Sensory analysis favored cheese made with the Mise94 strain, with grape pomace enhancing functional appeal while minimally impacting flavor [128]. These findings emphasize the potential of grape pomace as a functional ingredient that improves the nutritional profile and antioxidant capacity of cheeses.

Grape pomace fermentation is recognized as a technique to enhance the functionality of phenolic compound extracts by imparting prebiotic properties. The use of grape pomace in food preparations has been shown to facilitate the fermentation process of *L. acidophilus* and *S. thermophilus* by enhancing lactic acid synthesis and reducing fermentation time [129]. Furthermore, yogurts enriched with pectin extracted from grape pomace presented higher probiotic survivability, lower syneresis, and superior storage-dependent sensory attributes during 21 days of storage [130]. Unfermented grape pomace from various cultivars was incorporated into whole yogurt at a concentration of 60 g/kg. The analyses revealed a significant increase in antioxidant content compared to the control yogurt, which did not contain grape pomace. Microbiological analyses reported the survival of starter strains, *Streptococcus thermophilus* and *Lactobacillus delbrueckii* subsp. *bulgaricus* in the presence of grape pomace, with no negative effects. Nevertheless, the organoleptic profile of yogurt enriched with grape pomace had lower acceptability scores than the control yogurt, indicating a need for further improvement. Grape pomace significantly increased antioxidant activity in fermented milk, and fermented milk containing *L. rhamnosus* imparted good flavor, color, sustainability, and overall acceptability [12]. Furthermore, the prebiotic effects of grape pomace polyphenols were investigated in combination with probiotic *Lactobacillus* sp. for their anti-inflammatory properties; synergistic effects between the prebiotics and probiotics were revealed [131]. Grape pomace extracts fermented by fungal-produced hydrolytic enzymes have shown prebiotic activity by promoting *L. casei* growth [132]. In addition, Tseng and Zhao [11] described the use of grape pomace extracts as alternative sources of antioxidant dietary fiber to delay lipid oxidation in yogurt and salad dressings. They reported that the fortified products were mostly acceptable to consumers based on the sensory study.

The encapsulation of phenolic compounds is known to enhance their bioaccessibility and bioavailability. Therefore, the effects of adding micro-encapsulated pomace extract to coconut water on the growth of probiotic bacteria were evaluated [133]. The results indicated a positive effect on the growth of *Lactobacillus* and *Bifidobacterium* spp., with no negative impact on the sensory qualities, such as aroma and flavor. At concentrations up to 10%, grape pomace powder and extracts were added to apple and orange juice, exhibiting antifungal activity against *Zygosaccharomyces rouxii* and *Z. bailii* [81].

To prevent food spoilage due to bacterial proliferation (such as *Streptococcus* spp., *Lactobacillus* spp., *Aeromonas* spp., and *Leuconostoc* spp.), researchers have focused on natural antimicrobial components. A study indicated that the addition of grape seed extract inhibited the lipid hydrolysis processes in the dry-fermented pork neck, reduced the free fatty acid content, and reduced the number of *Enterobacteriaceae* in the meat product but did not limit the growth of probiotic bacteria [134]. Extracts of grape pomace and grape seeds were shown to extend the shelf life of lamb meat after a week of refrigerated storage [135]. The present antioxidants reduced lipid peroxidation and meat discoloration by donating hydrogen and sequentially quenching radicals [135]. In the fish industry, Cilli et al. [136] explored the incorporation of grape pomace flour at 1% and 2% concentrations in salmon burger formulations to enhance their stability and functional properties. The addition of grape pomace flour significantly reduced lipid oxidation during storage, with 2% showing the greatest antioxidant effect, comparable to the synthetic antioxidant butylated hydroxytoluene. The flour also enriched the burgers with dietary fiber and bioactive compounds, improving their nutritional profile. While grape pomace flour slightly darkened the burger color, sensory evaluations indicated general consumer acceptance.

The European Food Safety Authority (EFSA) stated that anthocyanins from grape pomace can now be used as food coloring in beverages, marmalades, candies, and ice creams in order to replace synthetic dyes [137]. Enocyanin, known as E163 in the food industry, is another naturally occurring color derived from grape skin anthocyanins [137]. Trentin et al. [138], examined the use of grape pomace as a source of anthocyanin-rich extracts to be incorporated into gummy candies as natural colorants and antioxidants. Using a natural eutectic solvent consisting of choline chloride, as well as acetic and citric acids, the anthocyanin extract achieved a high yield of 60 µg cyanidin-3-glucoside equivalent per gram of dry grape pomace and exhibited significant antioxidant activity. The study incorporated 2.5% to 10% of this extract into the candy formulations, resulting in an enhanced pink color and improved antioxidant properties. Additionally, the study measured moisture content and water activity, with the moisture content ranging from 23.7% in the control sample to 30.2% in the extract-enriched gummies, an essential parameter for the quality, sensory appeal, and shelf life. Shelf life was also examined, revealing that the extract-enriched gummies had extended durability. Fungal growth appeared after 20 days in the control sample and after 40 days in the highest extract concentration. Gummies with acetic acid-based eutectic mixtures showed no fungal growth for up to 60 days, demonstrating improved stability and longer shelf life.

The incorporation of grape pomace extract (GPE) into food packaging is an innovative solution aimed at reducing the environmental impact of petroleum-based plastics while enhancing food safety and prolonging shelf life. In this context, the antimicrobial properties of grape pomace extracts were transferred successfully to jute natural fabrics through supercritical impregnation. This new active food packaging material displayed antibacterial capacity against *Escherichia coli*, *Staphylococcus aureus* and *Pseudomonas aeruginosa*. It showed that it has the potential to function as natural fiber active food packaging [139]. Following the same approach, Raschip et al. [140] incorporated GPE from Feteasca Neagra and Merlot grape varieties into ice-templated 3D cryogels made from xanthan gum and polyvinyl alcohol. The study found that adding GPE reduced the cryogels’ swelling ratio and increased their hydrophobicity, especially at 30–40% extract concentrations, making them more suitable for food packaging. Additionally, the cryogels exhibited enhanced antioxidant and antimicrobial properties, effectively inhibiting the growth of *E. coli*, *S.* Typhimurium, and *L. monocytogenes*. Silva et al. [141] developed antimicrobial polypropylene films by incorporating grape pomace extract. These films exhibited significant antimicrobial activity, particularly against *E. coli* and *B. subtilis*, effectively inhibiting bacterial growth. Additionally, the films demonstrated low water vapor permeability, a critical feature for food packaging, as it helps to maintain the quality and freshness of packaged products by minimizing moisture loss. Nogueira et al. [142] examined the incorporation of GPE into arrowroot starch films. The results showed a noticeable change in physical properties such as increased thickness, water content, solubility, and water vapor permeability. The study also found that GPE acted as a plasticizer, reducing polymer chain interactions and enhancing film solubility, which is particularly beneficial for edible films.

### 5.2. Cosmetic Industry

Grape pomace extracts can be considered essential ingredients in cosmetic product formulations (lotions, emulsions, liquid gel-based serums, creams, and/or toothpaste) due to their high levels of bioactive compounds with skin depigmenting, anti-aging, and photoprotective properties.

Reis et al. [143] revealed a novel *V. vinifera* extract that acts on multiple targets related to skin aging, proposing it to be a promising anti-aging active ingredient candidate that should be further investigated in clinical trials. Cornacchione et al. [144] investigated the antioxidant properties of *Vitis vinifera* shoot extract, focusing on its effects on human keratinocytes and photoaged skin. In vitro, the extract demonstrated stronger antioxidant activity than vitamins C and E, effectively reducing reactive oxygen species (ROS) generated by hydrogen peroxide. In vivo trials involved formulations with Sarmentine (*Vitis vinifera* extract) and RonaCare Hydroine, tested on 27 subjects in a randomized, single-blind trial. The combination showed a greater antioxidant effect compared to Sarmentine alone. A clinical study with 60 female participants further revealed that a serum containing these ingredients improved photoaged skin, with enhancements in firmness, hydration, and texture after 4 weeks. Adding cream to the serum amplified these benefits. Recently, Wasilewski et al. [145] developed shower gels with 10–20% grape pomace extract solutions. The incorporation of these extracts endowed the gels with potent antioxidant properties, improved hydration, and reduced irritation, making them gentler than traditional formulations. These gels maintained functional integrity with stable viscosity and effective foaming while gaining consumer appeal through a natural red hue provided by the extracts. Additionally, Salem et al. [146] focused on Marselan grape seed extract, formulating creams with concentrations of 2–5%. The extract, rich in catechins, offered significant antioxidant protection against oxidative stress. Stability tests over 4 months demonstrated that the creams remained stable under varying conditions, with optimal results at 4 °C. The 5% concentration provided the best balance of stability and antioxidant efficacy, maintaining effectiveness across all storage conditions.

Another study assessed the safety and efficacy of a sunscreen system enriched with grape pomace extract (*Vitis vinifera* L.) [147]. Phenolic compounds from the grape pomace were recovered via percolation, and two formulations were developed: one with UV filters alone and another combining UV filters with 10% (*w/w*) grape pomace extract. The addition of grape pomace extract significantly boosted antioxidant activity and enhanced UVB protection. The extract-containing formulation delayed erythema 21% more than the UV-filter-only product, extending the time to induce skin redness by approximately 21%. These results indicated a synergistic effect between the grape pomace phenolic compounds and UV filters. Both formulations were deemed safe for use. Similarly, Yarovaya et al. [148] explored the effects of grape seed extract (GSE) on skin fibroblasts exposed to UVA light and its role in enhancing photostability when incorporated into sunscreen formulations. In vitro results showed that GSE exhibited significant antioxidant properties. Furthermore, when included in sunscreen formulations with standard UV filters, GSE significantly improved the photostability of the products, enhancing their UV protective properties under UV exposure, working synergistically with traditional UV, while preserving the cosmetic appeal of the formulations. Along the same lines, grape pomace extract, rich in catechin, quercetin, fisetin, and gallic acid, was incorporated into liposomes and phospholipid vesicles, which were modified using glycerol and MONTANOV 82^®^ (a surfactant), or a combination of both [149]. The vesicles demonstrated high loading efficiency, surpassing 100%, and exhibited enhanced antioxidant activity, increasing from 84% to 89%. Additionally, the grape pomace extract-loaded vesicles showed excellent biocompatibility and effectively protected 3T3 cell fibroblasts from oxidative damage.

Moreover, some cosmetic products containing grape pomace extract are already on the market, such as facial scrubs with grape seed oil (BioAroma Natural Products, Greece), sunscreen, skincare products, anti-aging moisturizers, anti-aging eye creams, face serum to treat dark spots, e.g., from Caudalíe (France), day creams/night creams, and grape pomace scrub kits (Alexandria, VA, USA). This particular grape pomace scrub kit has recycled pomace (29%) that helps repair human skin cells.

### 5.3. Pharmaceutical Industry

Grape pomace, whether dried and lyophilized, or in unfermented, semi-fermented, or fermented form, contains numerous nutraceutical compounds, mainly polyphenols. Nutraceuticals are typically consumed in pharmaceutical forms, such as pills, capsules, tablets, powders, and vials. Recently, a study by Schiano et al. [150] evaluated maltodextrinated grape pomace extract (MaGPE) as a nutraceutical adjunctive therapy for managing non-proliferative diabetic retinopathy (NDR) by reducing oxidative stress and improving retinal health. Conducted at the University Magna Graecia, Italy, between December 2020 and September 2022, the clinical trial involved 99 patients with NDR and center-involved diabetic macular edema (DME) unresponsive to dexamethasone injections. MaGPE, derived from Aglianico cultivar grape pomace and processed by MBMed Company (Turin, Italy), was prepared via water extraction and spray-drying with maltodextrins (40–70%). The extract was standardized for high antioxidant content and confirmed through HPLC-DAD. Patients were divided into two groups: one received 400 mg MaGPE tablets twice daily, and the other received equivalent doses of maltodextrin (placebo). After six months, the MaGPE group demonstrated significant increases in best-corrected visual acuity (BCVA), reductions in central retinal thickness (CRT, stabilization, slight increases in vascular perfusion percentage (+3.0% at 3 months and +2.7% at 6 months), decreased reactive oxygen metabolite derivatives (from 1100.6 ± 430.1 UCARR at baseline to 930.6 ± 310.3 UCARR), and lower oxidized LDL levels (from 953.9 ± 212.4 µEq/L to 735.0 ± 213.7 µEq/L). The MaGPE group also showed reduced retinal swelling and vascular leakage, improved retinal vascular health, and preserved vision. The treatment was well-tolerated, with no significant adverse effects reported.

An additional promising application of grape pomace in the pharmaceutical industry is as a vehicle for drug delivery systems. There are many dietary supplements based on grape seed and grape extract in the market as a result of their hypoglycemic, antioxidant, and hypolipidemic properties [67]. Products based on grape pomace are already commercialized as supplements in powder or capsule forms [151,152]. Dietary fiber from grape pomace has also been used as a supplement with potential prebiotic functions, reducing rancidity in seafood during ice storage [153]. Oligomeric proanthocyanidins from grape seeds have been supplemented to patients at doses of 150 mg/day in a randomized double-blind clinical design, demonstrating improvements in the antioxidant status and the serum lipid profiles of patients with chronic obstructive pulmonary disease [154]. Activ’Inside^®^ is another supplement available on the market that contains phenolic compounds from grape pomace. It is used to improve cognitive performance and help in weight management. Kalli et al. [67] reported on other available dietary supplements such as the Lamberts^®^ super strength antioxidant complex, Solgar grape seed extract 100 mg, and LifeExtension^®^ resveratrol with pterostilbene. One Lamberts^®^ super strength antioxidant complex tablet contains 10 g of oxygen radical absorbance capacity units from a mixture of green tea, grape seed, rosemary, and oregano extracts. As for Solgar^®^ grape seed extract, 100 mg contains 95% phenolic compounds, including monomeric polyphenols, oligomeric proanthocyanidins, and organic acids. LifeExtension^®^ resveratrol with pterostilbene includes synergistic phytonutrients such as *trans*-pterostilbene and quercetin, which can enhance resveratrol’s effects.

### 5.4. Other Grape Pomace Applications

Apart from the mentioned applications, grape pomace from wine industries can be used directly or treated with solid-state fermentation to generate protein-rich biomass, which can be added to animal diets. In lamb diets, oat bran and wheat bran were replaced with sun-dried grape pomace (12.2%) without affecting the meat quality [155].

Oliveira et al. [156] explored the potential of utilizing by-products from the wine industry as sustainable sources for pellet production, finding promising characteristics for energy recovery.

Low-temperature hydrothermal treatment of grape pomace has been found effective for obtaining raw material for biorefinery processes, rendering it an appropriate feedstock through the optimization of soluble sugar content [157]. Furthermore, anaerobic digestion produced methane from grape pomace, pulp, and seeds, with the pulverization of substrates enhancing degradability by up to 22% [6]. Da Ros et al. [30] found that anaerobic treatment of wine lees, grape pomace, and grape stems significantly increased the production of biogas, especially from wine lees and grape pomace.

Another application for grape pomace involves composting. Martínez Salgado et al. [158] assessed the evolution of chemical, microbiological, biochemical, and phytotoxicity parameters during the composting processes of nine open piles of grape pomace, goat, and horse manure. The compost materials obtained from grape pomace and goat or horse manure could be used as organic fertilizers or amendments, ensuring safety in terms of microbiology and phytotoxicity [158]. Another study explored the combined approach of the solid–liquid extraction of polyphenols from ground grape pomace using a hydroethanolic mixture; this was followed by composting the exhausted pomace [159]. The composting process was unaffected by extraction but was enhanced by the reduction in pomace particle size, improving final biostability and humic acid content [159]. Additionally, nine composting experiments with grape skins were conducted in laboratory reactors under varying initial moisture levels and airflow rates [160]. The results indicate that grape skin waste can be effectively converted into compost through an eco-friendly process, with the initial moisture content and aeration rate acting as important factors in the process efficiency expressed by organic matter content [160].

To overcome the inefficiency of the conventional methods used for treating heavy metal wastewater contamination, various agricultural wastes have been tested for this purpose as cost-effective processes. Although further studies are needed to validate the process, grape pomace was recently tested as a green adsorbent for removing lead from contaminated effluents, with maximum lead adsorption capacities of 40 mg/g and 64 mg/g for Merlot and Sauvignon Blanc grape pomaces, respectively, at pH 5.5 and 22 °C [161].

These grape pomace applications demonstrate its wide range of uses in the industrial sector, with technological developments focusing on harnessing the beneficial properties of this residual material.

## 6. Conclusions

Grape pomace is produced in high quantities globally, posing serious pollution concerns when disposed of in the environment. Alternatively, grape pomace can potentially be utilized to extract value-added compounds through green processing and biotechnological innovations. These compounds offer long-term health benefits, cater to consumers’ environmental concerns, and serve as inexpensive ingredients for the food, nutraceutical, pharmaceutical, and cosmetic industries. In the process approach, simple and efficient extraction and purification processes should be preferred for easy scale-up and economical production, to improve the recovery of bioactive compounds and preserve their integrity and commercialization potential. Further research should be carried out on the potential applications of grape pomace extracts or isolated compounds. It is important to determine the optimal amounts of bioactive compounds for the incorporation in products to provide functional benefits without negatively impacting sensory characteristics or desired effects, or to understand consumer response. Scaling up these processes and studying life-cycle analysis and techno-economic aspects are essential to determine their commercial feasibility, as few studies have addressed the cost implications of using grape pomace. In addition, its seasonal availability should be considered for overall industrial production. In summary, the valorization of grape pomace represents a viable strategy for advancing sustainable resource management and waste reduction within the winemaking industry. By converting these by-products into valuable resources, wineries can foster a more circular economic model, generate additional revenue streams, and support the development of eco-efficient products. This paper supports the integration of by-product utilization in winemaking processes to promote sustainability across the industry and extend these practices beyond it.

## Figures and Tables

**Table 1:** Physicochemical composition of grape pomace, as obtained from assorted studies.

Chemical Composition	Specie	Value	References
Ash (g/100 g DW)	*Vitis genus*	4.52 ± 0.07	[9]
	*Vitis vinifera*	5.14	[27]
		4.65–5.07	[11,28]
	----	2.53–6.17	[21]
		3.25	[29]
Arabinan (% DW)	*Vitis genus*	0.61 ± 0.04	[9]
Biochemical Methane Potential (L_N_CH4/kg Volatile Solids)		116–360	[30,31]
Carbohydrates (g/100 g DW)		29.2	[28]
Cellulose (g/100 g DW)		9.2–14.5 7–9	[32] [6,27]
Chemical Oxygen Demand (g O_2_/kg *w/w*)	*Vitis vinifera*	223–610	[6,30]
Energy value (Kcal/100 g)	*Vitis vinifera*	224	[28]
Fructose (g/100 g DW)	*Vitis genus* *Vitis vinifera*	1.09 ± 0.05 8.91 ± 0.08	[9] [28]
Galactan (% DW)	*Vitis genus*	0.79 ± 0.01	[9]
Glucan (% DW)	*Vitis genus*	8.04 ± 0.42	[9]
Glucose (g/100 g DW)	*Vitis genus* *Vitis vinifera*	0.21 ± 0.01 7.95 ± 0.07	[9] [28]
Hemicellulose (g/100 g DW)		4.0–10.3 6–22	[32] [6,27]
Lignin (g/100 g DW)	*Vitis genus*	44.46 ± 0.02	[9]
		11.6–17.2	[32]
		11–23	[6,27]
Lipids (g/100 g DW)	*Vitis vinifera*	8.5	[27]
		8.16–11.09	[11,28]
Mannan (% DW)	*Vitis genus*	0.57 ± 0.01	[9]
Pectin (% DW)	----	5.4–5.7	[32]
pH	*Vitis vinifera* ----	3.94 3.34	[6] [27]
Protein (g/100 g DW)	*Vitis genus*	12.68 ± 0.12	[9]
		7.0–14.5	[32]
		14.17 ± 0.08	[27]
	*Vitis vinifera*	8.49–10.32	[11,28]
		5.38–12.34	[21]
Sucrose (% DW)	*Vitis genus*	0.12 ± 0.01	[9]
Sugar (g/100 g DW)	*Vitis vinifera*	3.89–16.86	[11,28]
	*----*	1.38–77.53	[21]
		15–33	[3]
Total dietary fiber (g/100 g DW)	*Vitis vinifera*	65.56 ± 0.83	[27]
		46.17–61.32	[11,28]
	*----*	17.28–56.31	[21]
		19–38	[3]
Total Solids (g/ kg) DW	*Vitis vinifera*	371 ± 51	[30]
		434 ± 5	[6]
Volatile Solids (g/ kg)	*Vitis vinifera*	371 ± 5	[6]
		334 ± 30	[30]
Xylan (% DW)	*Vitis genus*	6.23 ± 0.13	[9]
Catechin (mg/g DW)	*Vitis vinifera*	9.9–16.1	[33]
		0.799–1.973	[34]
		3.387	[35]
		0.670	[36]
Epicatechin (mg/g DW)	*Vitis vinifera*	1.6–2.5	[33]
		0.890	[36]
Quercetin (μg/g DW)	*Vitis vinifera*	46.9–162.3	[34]
		557.3 ± 83.9	[35]
Gallic acid (μg/g DW)	*Vitis vinifera*	607–729.2	[34]
		252.8 ± 18.5	[35]
		116 ± 2	[36]
Hydrolysable tannins (mg TAE/g DW)	*Vitis vinifera*	3.7–5.3	[34]
Resveratrol (mg/g DW)	*Vitis vinifera*	0.0050–0.0069	[33]
Syringic acid (μg/g)	*Vitis vinifera*	1731.7 ± 156.3	[35]
Total anthocyanin content (mg cyd3-gluE/g GPE)	*Vitis vinifera*	48.29 ± 1.2	[16]
		1.1–1.4	[33]
		0.002–3.15	[20]
		1.31 ± 0.4	[28]
	*Vitis genus*	0.8–0.9	[9]
Total flavonoid (mg CE/g DW)	*Vitis vinifera*	26.32–37.98	[16]
		9.87–32.2	[20]
	*Vitis genus*	10.47–50.32	[9]
Total phenolic content (mg GAE/g GPE)	*Vitis vinifera*	511.11 ± 6.6	[16]
		24.5–30.4	[37]
		3.75–465.3	[38]
		86.3–117.8	[33]
		10.4–64.8	[20]
		40.5–60.1	[34]
		165.7–196.2	[35]
		67.74 ± 6.91	[11]
	*Vitis genus*	10.43–72.68	[9]
Total procyanidin (mg PB2E/g DW)	*Vitis genus* *Vitis vinifera*	7.68–11.30 7.68–55.3	[9] [20]
Procyanidin B2 (μg/g DW)	*Vitis vinifera*	308.3–1071	[34]
Vanillic acid (μg/g)	*Vitis vinifera*	15 ± 2	[36]
Vitamin C (mg AAE/g)	*Vitis vinifera*	26.25 ± 0.01	[22]
Vitamin E (mg/kg)	*Vitis vinifera*	5.00	[22]

AAE—ascorbic acid equivalent; CE—catechin equivalent; DW—dry weight; GAE—gallic acid equivalent; PB2E—procyanidin B2 equivalent; TAE—tannic acid equivalent; GPE—grape pomace extract; cyd3-gluE—cyanidin-3-glucoside (cyd-3-glu) equivalent.

**Table 2:** Advantages and disadvantages of conventional and modern extraction techniques.

Method	Advantages	Disadvantages
Conventional [15,19,40]	- Commonly used - Accessible - Satisfactory recovery rates - Easiest methods	- Chances of impurities (low extract quality) - Introduction of analytical errors - Non-eco-friendly (toxic solvents) - Slow mass transfer - Fail to meet the economic aspects of production
Supercritical Fluid Extraction [15,19]	- High extraction rate - Automatic system - No requirements for additional filtration or centrifugation - Possibility of reusing CO_2_ - Extraction of thermolabile compounds at lower temperatures - Nontoxic, non-flammable, nonexplosive, and considered a food-grade solvent (GRAS) - Exceptional mass transfer properties - Environmentally friendly and energy-efficient process	- Excessive cost of equipment - Risk of losing volatile compounds - High pressures increase the process costs - Insolubility of polar extracts in the CO_2_ mobile phase - Onerous operating conditions
Ultrasound-Assisted Extraction [15,46,47]	- High mixing and extraction efficiency - Selective extraction - Fast rate of extraction - Inexpensive - Effective for thermolabile compounds (lower operating temperature) - Less amounts of solvent and energy used	- Linked to the plant matrix nature - Requires filtration - Uneven distribution of ultrasound energy may lead to a lack of uniformity - More solvent required
Microwave-Assisted Extraction [14,15]	- Shorter extraction times (3–30 min) - Less consumption of solvents - High extraction yields - Speed uniformity of heating - Selective heating - Can be turned on or off instantly - Lower risks - Preservation of colors, flavors, and nutrients	- Not particularly effective for non-polar target compounds or solvents - Requires engineering expertise to comprehend and mitigate uneven heating or thermal runaway - High equipment costs - Requires filtration step
High-Voltage Electric Discharge (HVED) [49,50]	- Fewer energy requirements - High extraction efficiency	- Possible degradation of extraction compounds - Limited lifetime of electrodes - Poor selectivity
Deep Eutectic Solvents (DESs) [15,47,49]	- Low toxicity and price - High extraction yields - Biodegradable - Capable of adjusting viscosity, polarity, and density	- Need for a filtration step - Viscosity or high-density mixture

**Table 3:** Patents currently available in the WIPO-PATENTSCOPE database related to the industrial applications of grape pomace.

Patent ID	Title	Coverage	Focus	Publication Date	Jurisdiction
WO2014013122	Polyphenol extract from white grape residue	This invention describes an environmentally friendly method for obtaining a polyphenol-rich extract from white grape pomace, for several applications.	Several industries (food, cosmetic, and pharmaceutical)	23 January 2014	Spain
CN104798854	Method for making grape pomace dietary fiber nutritious biscuits	The invention describes a method for producing nutritious biscuits using grape pomace. The final product retains the characteristic texture of traditional biscuits while incorporating the dietary fiber from grape pomace, enhancing the nutritional value and health benefits.	Food industry	11 May 2015	China
CN114601152	Actinidia arguta product rich in dietary fibers and lactic acid bacteria and preparation method of Actinidia arguta product	This patent involves processing grape pomace and other fruit by-products during the production of dietary fiber-rich Actinidia arguta products.	Food industry	10 June 2022	China
CN114951239	Method for preparing derivative raw material by recovering grape pomace in the wine-brewing process	This invention covers a method for extracting and stabilizing grape pomace to be used as a stable raw material in food industry applications.	Food industry	30 August 2022	China
WO2024065062	Process for converting pomace derived from wineries into a food ingredient and product	This invention presents a process to transform grape pomace into a nutrient-rich food ingredient, which can be used as an ingredient in foods as a flavor, texture, and color enhancer, to mask bitter flavors or off-notes, as a preservative, to fortify processed foods.	Food industry	4 April 2024	Canada
RU0002775316	Method for production of pastilles with functional properties	The invention describes a method for producing pastilles with functional properties, incorporating grape powder derived from grape pomace, apple puree, and lactic acid.	Nutraceutical	29 June 2022	Russian Federation
WO2023106937	Grape skin compositions, compounds, and methods of preparation and use	The disclosure covers grape skin concentrates and their compositions, along with methods for preparing and using them. These concentrates are specifically used in the preparation of various beverages, including fermented drinks.	Nutraceutical	15 June 2023	New Zealand
WO2024061367	Novel compound, pharmaceutical composition thereof, and use thereof	This patent pertains to the utilization of bioactive compounds derived from grape pomace in innovative pharmaceutical formulations aimed at treating neurodegenerative and cardiovascular or cerebrovascular diseases.	Pharmaceutical	28 March 2024	China
CN114747424	Lentinus edodes culture medium with anti-aging effect	The invention introduces a Shiitake mushroom culture medium with anti-aging benefits. This culture medium is composed of various ingredients, including grape pomace as one of its key components. Grape pomace helps improve the anti-aging properties of the mushrooms.	Cosmetic	15 July 2022	China
WO2024026416	Botanical extract blend for use in skincare	This patent focuses on the development of a botanical extract blend for skincare products, utilizing decolorized grape pomace extract, known for its antioxidant and skin-soothing properties.	Cosmetic	1 February 2024	United States of America
WO2024142004	“Oleolyte” of lyophilized apple or grape pomace with a high content of ursolic acid	The invention outlines a process for extracting ursolic acid from lyophilized apples or from lyophilized grape pomace, particularly from the Fiano di Avellino DOCG variety to create an “oleolyte”. This “oleolyte” is then utilized in cosmetic formulations.	Cosmetic	4 July 2024	Italy
CN114568584	Preparation method of grape branch-coated silage	This patent outlines a method for preparing silage from grape branches, preserving the biological properties for livestock feed.	Animal feed	25 March 2022	China
